# Hare-Lip in France

**Published:** 1851-03

**Authors:** 


*Hare-Lip in France.*—It is, perhaps, not generally known in this country, that French surgeons are in the habit of operating upon children for this deformity a few days after birth. The justly celebrated Paul Dubois is a great advocate for this practice, which, it is contended, is extremely advantageous, as children at that early period, do not struggle: union is obtained very rapidly, and the little creatures cry but little, take the breast well, and do not seem to suffer much. M. Guersant, surgeon to the children's hospital, stated the other day, before the surgical society of Paris, that out of seven children operated upon almost immediately after birth, he failed only once; whilst out of the same number of children one month old, he failed five times. He attributes this disproportion to the fact, that newly-born children can do without the breast during four days; ( ? ) they thereby are not apt to tear open the sutures by efforts at suction.—*Lancet*, from *Ib.*

